# Impact of exercise and fasting on mitochondrial regulators in human muscle

**DOI:** 10.1515/teb-2024-0014

**Published:** 2024-08-19

**Authors:** Eveline S. Menezes, Hashim Islam, Benjamin B. Arhen, Craig A. Simpson, Chris McGlory, Brendon J. Gurd

**Affiliations:** School of Kinesiology and Health Studies, Queen’s University, Kingston, ON, Canada; School of Health and Exercise Sciences, University of British Columbia – Okanagan, Kelowna, BC, Canada

**Keywords:** food deprivation, caloric restriction, muscle remodeling, aerobic exercise, transcriptional regulators

## Abstract

**Objectives:**

To investigate the impact of acute energetic stress (acute HIIE and fasting) on *ERRγ*, *PPARβ*, *NR1D1*, *NR4A1*, and *TFEB* in human skeletal muscle.

**Methods:**

The current study performed secondary analyses using muscle biopsy samples from two previously published studies: study 1) leg muscle biopsies from nine men and eight women were obtained pre and 3 h following acute high-intensity interval cycling exercise (HIIE); study 2) leg muscle biopsies were obtained from nine men pre-, during, and post-an 8 h fast with or without 2 h of arm ergometer exercise. RT-PCR was performed on samples from each study to determine the mRNA expression of *ERRγ*, *PPARβ*, *NR1D1*, *NR4A1*, and *TFEB*. Additionally, we retrieved data from meta-analyzed human muscle gene expression using the publicly available database MetaMex.

**Results:**

*PGC-1α* (p<0.01, d=1.98) and *NR4A1* (p<0.01, d=1.36) mRNA expression significantly increased while *TFEB* (p≤0.05, d=0.70) decreased following HIIE. Significant decreases in *NR4A1* and *NR1D1* mRNA expression were observed following an 8 h fast. Our MetaMex analyses revealed significant increases (p<0.05) in *PGC-1α* and *NR4A1* expression following aerobic and resistance exercise, and in *PPARβ* expression following resistance exercise.

**Conclusions:**

Our data indicate that acute HIIE stimulates increases in *NR4A1* and *PGC-1α* and decreases in *TFEB* mRNA expression in human skeletal muscle. Additionally, a short term (8 h) fast reduced the mRNA expression of the transcriptional regulators *NR4A1* and *NR1D1* – potentially as a mechanism of decreasing mitochondrial biogenesis to reduce energy expenditure during a period of restricted energy availability.

## Introduction

Current dogma in muscle biology argues that exercise-mediated increases in DNA transcription and mRNA expression drive mitochondrial protein synthesis [1] (i.e. increases in mRNA result in proportional increases in translation and thus protein content). Under this dogma, the pre-transcriptional control of mitochondrial biogenesis by intramuscular signaling and the purported “master regulator of mitochondrial biogenesis” *PGC-1α* have been studied extensively. However, the prevailing view of *PGC-1α* as “master regulator” is inconsistent with observations that: 1) *PGC-1α* is dispensable for exercise-mediated mitochondrial biogenesis [2], and 2) many genes function as transcriptional regulators [3]. Thus, it appears that transcriptional control of gene expression is determined by a layered network of transcriptional regulators [4]. At present, relatively limited evidence is available regarding the expression of transcriptional regulators beyond *PGC-1α*, especially in human skeletal muscle.

Of the many proteins purportedly involved in the regulation of mitochondrial biogenesis we chose to focus on *ERRγ*, *PPARβ*, *NR1D1*, *NR4A1*, and *TFEB* in the current paper. These genes are implicated in the molecular control of mitochondrial biogenesis in overexpression and knockout models in muscle cells and rodent skeletal muscle [5], [6], [7], [8], [9], [10], [11], [12], [13], [14], [15], [16], [17], [18], [19]. The impacts of acute energetic stress – specifically acute High Intensity Interval Exercise (HIIE) and short-term fasting – on the expression *ERRγ*, *PPARβ*, *NR1D1*, *NR4A1*, and *TFEB* are underexplored in human skeletal muscle. HIIE is a known mediator of mitochondrial biogenesis in human muscle [20]. Further, although the impact of fasting on mitochondrial biogenesis is controversial in humans, fasting can activate a transcriptional response in rodent muscle [21]. In rat muscle food deprivation (i.e. short term fasting <8 h) activates AMPK [22], a response that may be augmented by elevated whole body energetic stress [23].

The effect of exercise on *ERRγ* expression in human muscle is controversial – some transcriptomic data support [24], and some refute [25] an upregulation following endurance exercise. Similarly, *PPARβ* mRNA expression is sometimes [25], [26], [27], [28] but not always [24, 29] exercise-inducible in human skeletal muscle. In gene array studies *NR4A1* is robustly upregulated following a variety of exercise stimuli [24, 25, 30], [31], [32], an effect mainly mediated by adrenergic stimulation [33]. To our knowledge *TFEB* expression has only been characterized using transcriptomics approaches in human skeletal muscle [24, 25] – both studies reported no impact of endurance exercise – while no studies have examined the effect of exercise on *NR1D1* expression.

Although information is available about the effects of exercise on the regulation of *ERRγ*, *PPARβ*, *NR1D1*, *NR4A1*, and *TFEB*, comparably little data exists on the impact of fasting on the expression of transcriptional regulators. Data from rodent models provides some indication that fasting may decrease *PPARβ* and increase *TFEB* expression [34, 35]. However, to our knowledge the impact of a single fasting period on the expression of *ERRγ*, *PPARβ*, *NR1D1*, *NR4A1*, and *TFEB* expression in human muscle is unknown.

**Figure 1:**
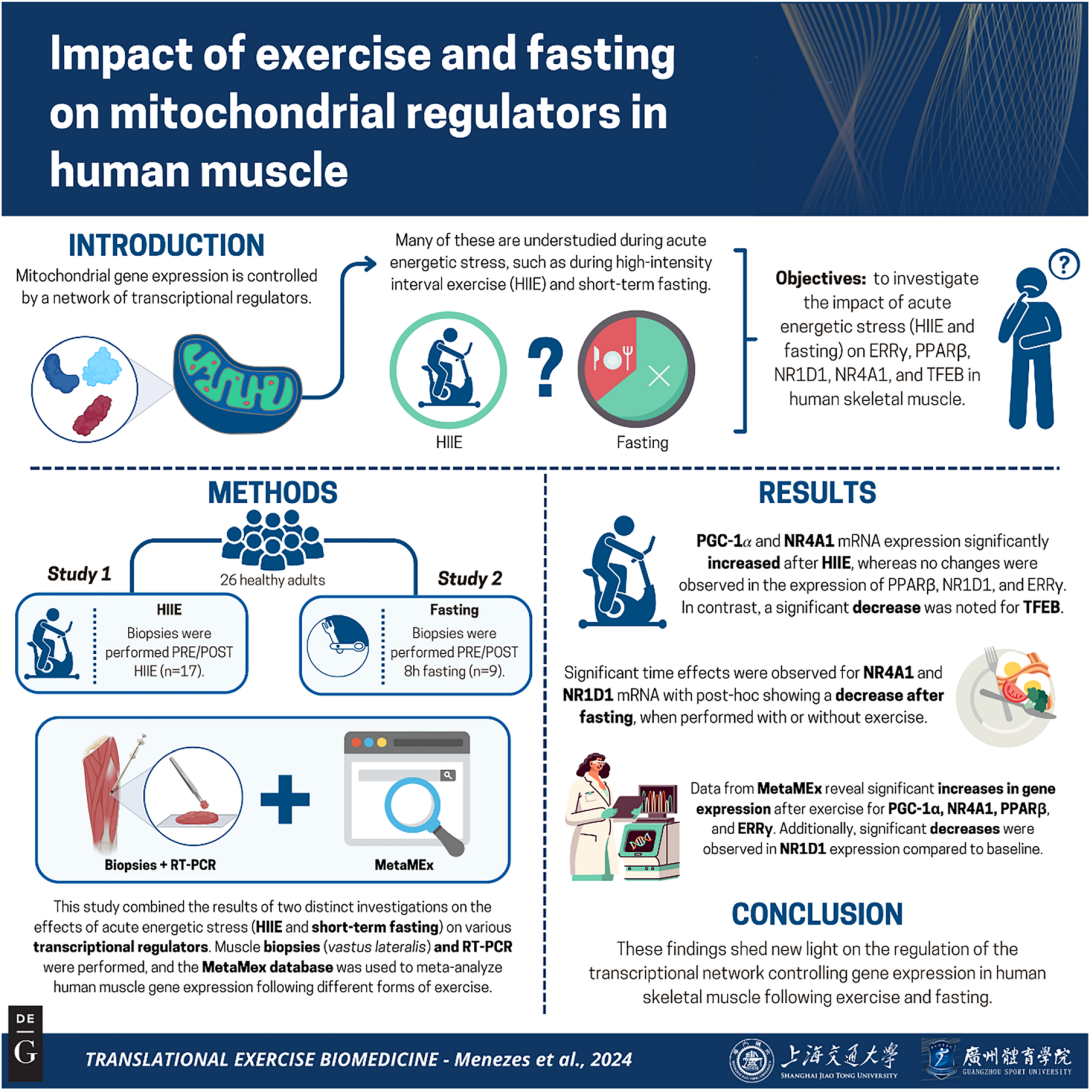
Graphical representation of this study. Key points: (1) The current study investigated the impact of energetic stress on understudied regulators of mitochondrial biogenesis. (2) Acute high intensity exercise alters the expression of NR4A1 and TFEB while an 8 hour fast suppressed expression of NR4A1 and NR1D1. (3) The results of this study contribute to our understanding of the layered network of transcriptional regulators that control of mitochondrial biogenesis. Figure created with BioRender. [Correction added after online publication, 20 November 2024: the original caption “Graphical representation of this article. Figure created with BioRender.” was updated as seen above.]

The purpose of this study was to examine the regulation of *ERRγ*, *PPARβ*, *NR1D1*, *NR4A1*, and *TFEB* mRNA following acute energetic stress (acute HIIE and an 8 h fast) in human skeletal muscle. We used two distinct approaches to achieve this purpose: 1) RT-PCR to examine changes in *ERRγ*, *PPARβ*, *NR1D1*, *NR4A1*, and *TFEB* gene expression following a single bout of HIIE and following an 8 h fast in the presence and absence of augmented energetic stress, and 2) we retrieved data from meta-analyzed human muscle gene expression available on MetaMEx (https://www.metamex.eu) [36] to determine the regulation of *ERRγ*, *PPARβ*, *NR1D1*, *NR4A1*, and *TFEB* in response to acute aerobic, resistance and HIIE exercise. To provide context for the changes in the genes explored in the current study, we present alterations in *PGC-1α* due to its importance in the control of mitochondrial biogenesis and purported interactions with the transcriptional regulators examined. The preliminary data we generated in this study will help advance our understanding of the mechanisms underlying the complex regulatory network involved in the regulation of skeletal muscle mitochondrial biogenesis. The summary of this article is presented in Figure 1.

## Methods

### Participants

The current study performed secondary analyses using real-time reverse transcription–polymerase chain reaction (RT-PCR) on muscle biopsy samples from two previously published studies [23, 37]. Seventeen (n=9 males; n=8 females) healthy young individuals volunteered to participate in protocol 1 (acute HIIE) [37], while nine healthy young males volunteered to participate in protocol 2 (fasting vs. fasting + exercise) [23]. Participant characteristics for both protocols are presented in Table 1. For both studies participants were included if they were recreationally active (i.e. self-reported involvement in ≤3 h of weekly moderate-to-vigorous intensity exercise), between 18 and 30 years of age, non-smokers, not taking any performance enhancing supplements and/or prescription medication intended for the treatment of cardiometabolic disease (e.g. metformin), not involved in a systematic training program aimed at improving specific aspects of cardiorespiratory fitness and/or muscular strength/endurance at the time of the study, body mass index <30 kg/m^2^, and did not have a history of cardiometabolic disease (e.g. diabetes, hypertension). Physical activity levels and readiness were assessed using a 7-day Physical Activity Recall Questionnaire (PAR-Q). Hormonal cycle phase and oral contraceptive use in female participants were not controlled. Both studies were approved by the Health Sciences Research Ethics Board at Queen’s University in accordance with the declaration of Helsinki and all participants provided verbal and written consent prior to any data collection. The original protocols were registered through the Open Science Framework for protocol 1 (https://doi.org/10.17605/OSF.IO/U7PX9) and through the ClinicalTrials.gov for protocol 2 (https://clinicaltrials.gov/ ID: NCT03811717).

**Table 1:** Participant characteristic summary of protocol 1 and 2.

	Females (n=8) [protocol 1]	Males (n=9) [protocol 1]	Total (n=17) [protocol 1]	Males (n=9) [protocol 2]
Age (yr.)	21.6 ± 2.26	22.40 ± 4.56	22.18 ± 3.52	21 ± 3
Body mass, kg	61.24 ± 11.2	84.01 ± 9.76^a^	73.29 ± 15.49	84 ± 11
Height, cm	167.19 ± 8.07	180.29 ± 4.74^a^	173.79 ± 9.50	183 ± 6
BMI, kg/m^2^	21.9 ± 3.3	26.1 ± 3.01^b^	24.1 ± 3.4	25.08 ± 3.67
VO2peak, ml/min/kg	36.3 ± 6.39	43.47 ± 7.44	39.88 ± 7.65	46 ± 7
HR average	151.67 ± 10.8	156.13 ± 9.3	153.8 ± 10	
HR max	187.33 ± 12.9	188.38 ± 9.3	187.8 ± 11	
Δ lactate	8.92 ± 3.1	13.80 ± 3.1^c^	11.2 ± 3.9	
RPE	19.33 ± 0.4	19.88 ± 0.4	19.9 ± 0.3	

Values presented as mean ± SD. ^a^Significant difference (p<0.001), ^b^(p=0.006), ^c^(p=0.005).

### Experimental design

**Figure 2:**
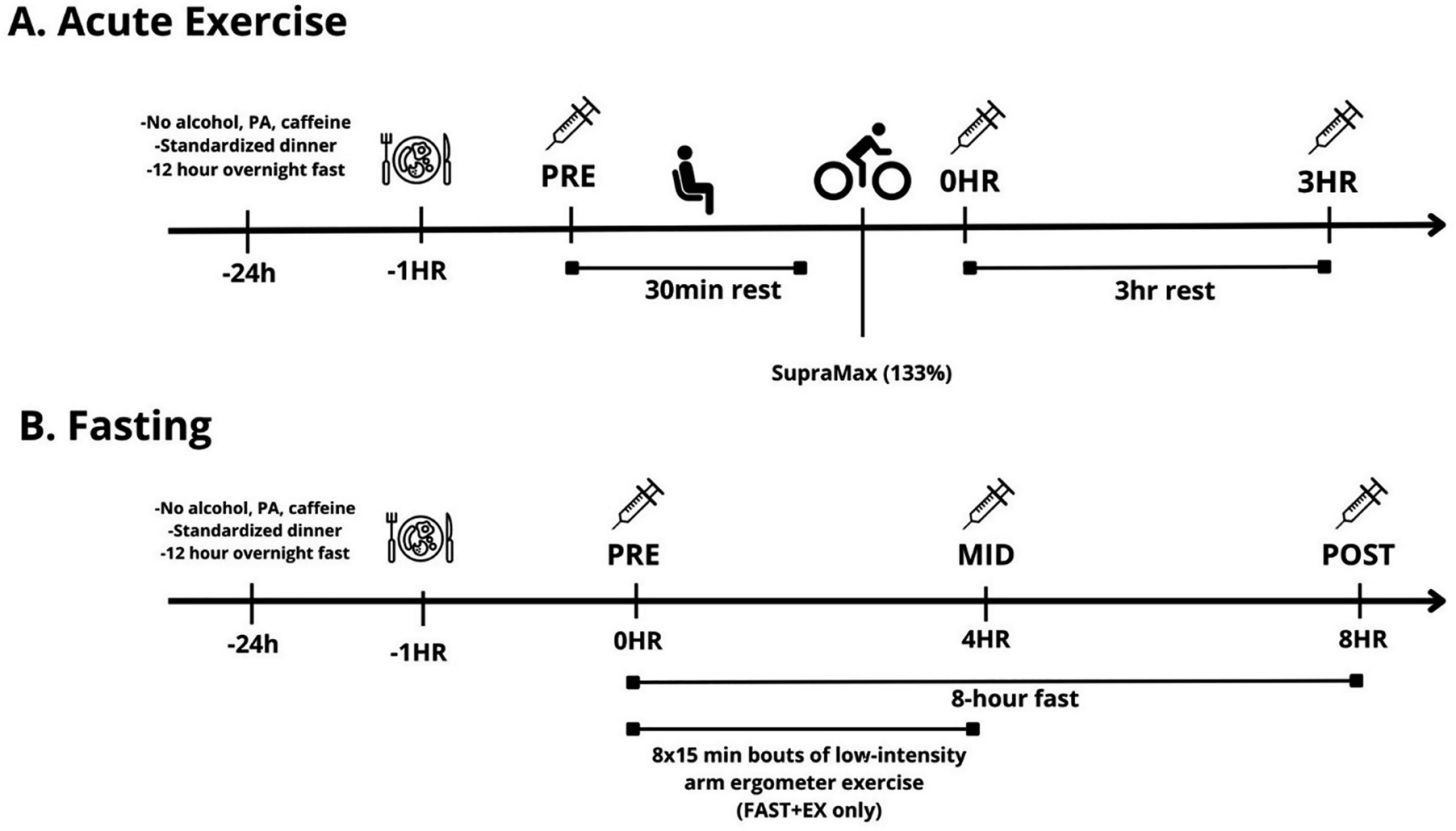
Experimental timeline. A. acute exercise; B. fasting.

Detailed experimental information for both protocol 1 (acute exercise) and protocol 2 (fasting vs. fasting + exercise) have been published previously [23, 37]. Details of protocol 1 are presented according to the CERT guidelines for reporting exercise trials (see Worksheet S4 in the Supplementary Document). For protocol 1 [37] participants completed a VO_2_peak test in the week preceding the first experimental visit. During two subsequent experimental visits, participants performed sessions of HIIE targeting either 100 % or 133 % of their peak aerobic power (WRpeak) in randomized order. Only tissue from the 133 % condition was utilized in the current analysis (see Figure 2-A for experimental timeline) because samples from the 100 % condition were allocated for a separate study to investigate distinct parameters. All interval sessions were performed individually on the same cycle ergometer (Monark, Ergomedic 874E, Varberg, Sweden) and supervised by the same investigator (qualified master’s degree student in Kinesiology, CPR certified). Adherence to exercise was measured and reported using logs, with 100 % session attendance as the criteria. Motivation strategies included vocal encouragement and participant-selected music playlists. Participants could progress to the next session upon successful completion of the first without adverse effects. The study did not include a home program or nonexercise components, and no adverse events occurred during the sessions. All HIIE sessions were conducted at Queen’s Muscle Physiology Lab (QMPL). All participants performed the same HIIE sessions, starting with a five-minute load-less warm-up followed by one-minute intervals at 80 RPM, separated by 1 min of load-less cycling at a self-selected cadence. During intervals, a load was added to achieve 100 % (max) or 133 % (supra) of peak aerobic power (highest 30-second power output from their VO_2_peak test). The intensities ensured a matched amount of external work in eight intervals for max and six for supra. Participants maintained 80 RPM, with additional intervals added if RPM fell below 80 to achieve the target work. The exercise intervention was delivered and performed as planned. Muscle biopsies taken before (pre) and 3 h (3 h) after exercise were snap frozen in liquid nitrogen and used to determine changes in mRNA expression.

For protocol 2 [23] participants completed two experimental sessions in a randomized and counterbalanced cross-over fashion (see Figure 2-B for experimental timeline). Experimental trials consisted of two supervised sessions: one 8 h fast performed with (FAST+EX) and one without (FAST) the addition of 2 h of low-intensity arm ergometer exercise (∼400 kcal of added energy expenditure completed during the first 4 h of the fast). Arm ergometer exercise was utilized to elevate whole-body energetic stress during the fasting period. Briefly, arm exercise consisted of 8 15 min intervals at 25 W separated by 10 min of seated rest. HR was recorded and was used to estimate total L O2 consumed based on the HR-VO_2_ relationship established during the incremental test. Total L O2 was converted to kcal assuming 5 kcal/L O2 consumed. Biopsies were obtained from rested (i.e. non-exercised) leg muscle before (pre), during (4 h) and after (8 h) each fast. In the fasting only session (FAST), participants rested in a seated position for the entire 8 h fast (except for tissue sampling time-points and washroom visits).

In both protocols an incremental step test (25 W/min) to volitional fatigue was completed on a cycle ergometer (Monark, Ergomedic 874E, Varberg Sweden) for the determination of peak oxygen uptake (VO_2_peak). Prior to starting exercise (protocol 1), or before starting to fast (protocol 2), participants consumed a standardized breakfast (bagel with cream cheese and orange juice [∼410 kcal, 10 g fat, 11 g protein]).

### Tissue sampling

All skeletal muscle biopsies were obtained using the Bergström muscle biopsy technique from lateral portion of the *vastus lateralis* under superficial local anesthesia (2 % lidocaine with epinephrine) as described in Islam et al. [23]. For both protocols biopsies were obtained from a single leg during each experimental visit. Muscle was snap frozen in liquid nitrogen immediately following each biopsy.

### Gene expression

RNA extraction, reverse transcription, and RT-qPCR were performed as we have done previously [23] on both data sets (protocol 1, Pre and 3 h samples; protocol 2, Pre, 4 h and 8 h samples). Following snap freezing in liquid nitrogen muscle samples were ground to a fine power using a mortar and pestle before being resuspended in a buffer containing guanidine thiocyanate, sodium citrate, sarkosyl and β-mercaptoethanol [38, 39]. Extracted RNA samples from acute HIIE and fasting protocols had an average 260:280 ratio of 2.00 ± 0.04 and 2.00 ± 0.02 (mean ± standard deviation [SD]), respectively. One microgram of RNA was reverse transcribed using the QuantiTect Reverse Transcription Kit (Qiagen, Mississauga, Ont., Canada) and mRNA levels were determined on an QuantStudio™ three system Real-Time PCR System (Thermo Fisher Scientific, Waltham, MA, USA). Forward and reverse primers sequences are provided in worksheet S2 in the Supplementary Document. Average primer set-specific efficiencies were *E*=1.95 ± 0.09 (mean ± SD). Results were analyzed according to the ΔC_q_ method using TATA-binding protein (TBP) as a housekeeping gene.

### Bioinformatic analyses

In addition to the secondary analyses described above, we conducted six bioinformatic analyses to examine changes in our genes of interest in a large dataset of acute aerobic, acute resistance, and acute HIIE studies. These analyses were completed using a gene expression database containing meta-analysis of skeletal muscle response to exercise (MetaMEx; https://www.metamex.eu) [36]. The following official gene symbols were used for the MetaMEx search: NR1D1, NR4A1, TFEB, PPARGC1A (for PGC-1α), PPARD (for PPARβ), and ESRRG (for ERRγ). Using MetaMEx we customized the search criteria to define the human population of interest. The search parameters were specified as follows: for sex, we included studies involving males, females, and those with undefined sex; for age, we included studies involving young, middle-aged, and elderly individuals; for fitness, we included sedentary, active, and athlete individuals; weight categories encompassed lean, overweight, obesity, and obesity class 3; muscle groups included vastus lateralis, biceps brachii, soleus, and gastrocnemius; and for health status, we selected studies involving only healthy individuals. All available timepoints (immediate, 1 h, 3 h, 4 h, 5 h, 6 h, 8 h, 18 h, 24 h, 48 h, and 96 h post exercise) were included. Data from time course analyses performed on all available data from healthy individuals were also extracted from MetaMEx.

### Statistical analyses

Statistical analyses were performed using GraphPad Prism version 9.4.1 for Windows, GraphPad Software, San Diego, California USA, www.graphpad.com. We used an independent T-Test to compare male and female participants’ characteristics from protocol 1 (see Table 1). The effect of HIIE (133 % WRpeak) on *PGC-1α*, *ERRγ*, *PPARβ*, *NR1D1*, *NR4A1*, and *TFEB* mRNA expression was compared using paired t-tests. Corresponding effect sizes were determined using an online effect size calculator (https://www.socscistatistics.com/effectsize/default3.aspx) and interpreted for within-subjects design as small (d=0.2) medium (d=0.5), and large (d=0.8). Correlation between changes in gene expression and physiological and psychological changes (HR average, HRmax, lactate, RPE) following exercise was assessed using Pearson (r) correlation coefficients. Pearson correlation coefficients were classified as very weak (<0.19), weak (0.20–0.39), moderate (0.40–0.59), strong (0.60–0.79), or very strong (>0.80).

In our original HIIE study, *a priori* sample size calculations were performed for the primary outcomes of between Max and Supra max intensities for *PGC-1α* mRNA (paired t-test; d=1.6, *α* error probability=0.05, 1-β error probability=0.8) and within participant pre/post changes in AMPK activation (p-AMPK and p-ACC; 2-way RM ANOVA; η2=0.0588, *α* error probability=0.05, 1-β error probability=0.8, correlation r=0.7). A sample size of 18 was chosen to provide statistical power to detect a large effect for *PGC-1α* expression and a medium effect for p-ACC/p-AMPK (for full details see [37]). Due to dropouts in our original study a final sample size of 17 (males n=9; females n=8) was utilized in the current analysis unless otherwise specified below.

The effect of fasting with and without augmented energetic stress on *PGC-1α*, *ERRγ*, *PPARβ*, *NR1D1*, *NR4A1*, and *TFEB* mRNA expression was examined using two-way (condition × time) repeated measures ANOVAs. Sphericity assumption for the two-way ANOVA was respected. Significant main effects and/or interactions were subsequently investigated using *Tukey’s* post hoc tests. Corresponding effect sizes were determined using an online effect size calculator \(Uanhoro, 2017, available online at: https://effect-size-calculator.herokuapp.com/.) and interpreted using partial eta squared (η2) values (small=0.0099; medium=0.0588; large=0.1379) [40]. All statistical analyses of mRNA for both protocols were performed on linear data (2^−ΔCq^ values) with *TBP* as a housekeeping gene [41]. Statistical significance was accepted at p*<*0.05.

In our original fasting study, the primary outcome measure was *PGC-1α* mRNA. However, due to the lack of prior human studies on skeletal muscle *PGC-1α* mRNA response to an 8 h fast (with or without exercise), a formal sample size calculation was not conducted and a sample size of 10 was considered sufficient to detect a medium effect size difference (Cohen’s f=0.25) with 80 % power at an alpha level of 0.05. This assumption considered a correlation of 0.85 among repeated measures, as calculated for a repeated measures ANOVA (within-between interaction) using G*power v3. Due to limited sample availability, a final sample size of nine was utilized in the current analysis.

## Results

### Participant characteristics

Seventeen participants (n=9 males; n=8 females) completed the acute HIIE prescribed in protocol 1. Nine participants completed all aspects of both experimental sessions for protocol 2. Participants characteristics for both protocols are presented in Table 1. Body mass (p<0.001), height (p<0.001), BMI (p=0.006), and delta lactate (p=0.005), in Study 1 were statistically different between male and female participants.

### Response to HIIE

**Figure 3:**
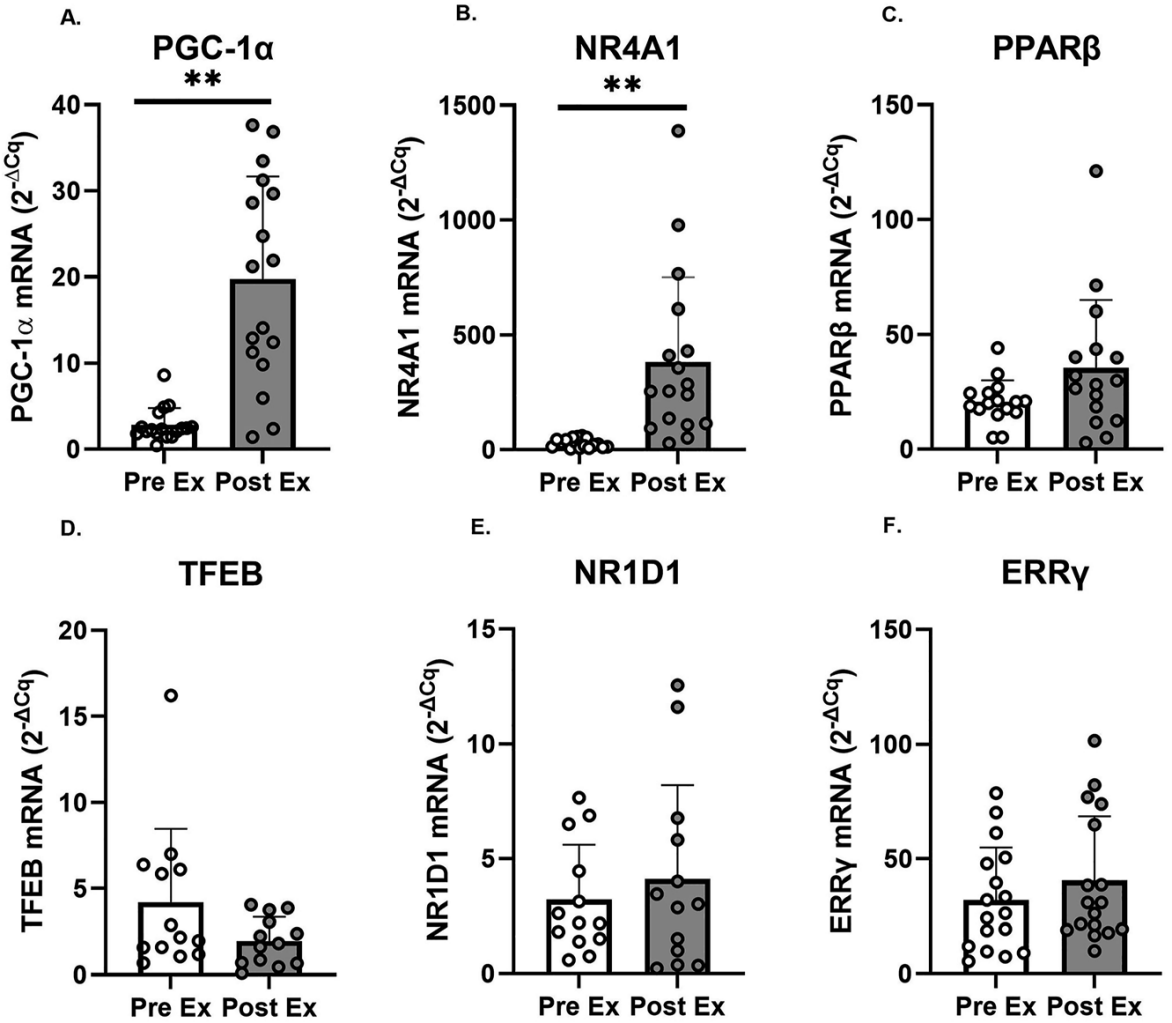
Changes in PGC-1α, NR4A1, PPARβ, TFEB, NR1D1, and ERRγ mRNA expression in the vastus lateralis before (pre ex; white boxes) and after (post ex; 3 h; grey boxes) an acute supramaximal exercise (n=17 for PGC-1α, NR4A1, and ERRγ; n=16 for PPARβ; n=13 for TFEB and NR1D1). Note: Symbols denote significant (**p<0.001) differences vs. PreEx. Individual data points representing each participant are superimposed on each bar graph, with open circles for PreEx and filled circles for PostEx.

Changes in the mRNA expression of regulators of mitochondrial biogenesis after acute HIIE are presented in Figure 3. *PGC-1α* (p<0.001, d=1.98) and *NR4A1* (p<0.001, d=1.36) mRNA expression significantly increased after HIIE. No significant changes were observed in the expression of *PPARβ* (p=0.104, d=0.67), *NR1D1* (p=0.419, d=0.27), and *ERRγ* (p=0.215, d=0.33). A significant decrease was observed for *TFEB* (p=0.053, d=0.70) after HIIE (see worksheet S3-Table A in the Supplementary Document). No strong or very strong correlations were observed between RPE and the gene expressions (all r<0.59); between HRmax and gene expressions (all r<0.59); between HR average and gene expressions (all r<0.59); or between delta lactate and gene expressions (all r<0.59). The expression levels *PGC-1α*, *NR4A1*, *ERRγ* were analyzed in a sample size of 17. However, due to limited sample availability, the analysis for *PPARβ* was performed on a sample size of 16, and *TFEB* and *NR1D1* on a sample size of 13.

### Response to fasting

**Figure 4:**
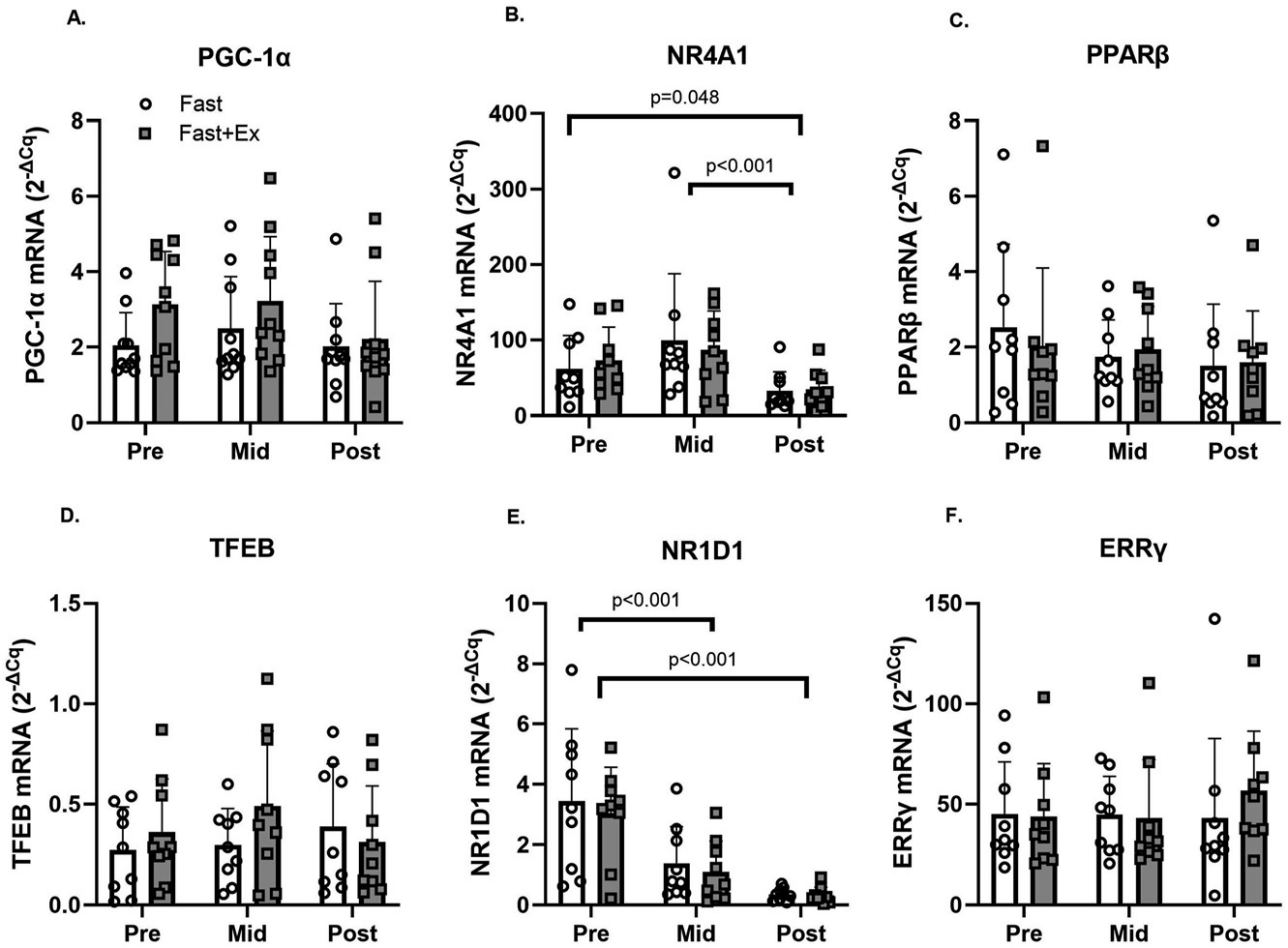
Changes in PGC1α, NR4A1, PPARβ, TFEB, NR1D1, and ERRγ mRNA expression in the vastus lateralis before (PRE), during (mid; 4 h) and after (POST; 8 h) an acute fast performed with (FAST+EX; grey boxes) or without (FAST; white boxes) 2 h of low-intensity arm ergometer exercise (n=9). Note: Significant main effects (two-way RM-ANOVA) are reported below graphs where appropriate. Individual data points representing each participant are superimposed on each bar graph, with open circles for FAST and filled squares for FAST + EX.

Changes in the mRNA expression are presented in Figure 4 and corresponding p-values and effect sizes are presented in worksheet S3 in the Supplementary Document. Significant time effects were observed for *NR4A1* and *NR1D1* mRNA. No other significant (p<0.05) main effects or interaction effects were observed (see worksheet S3-Table B in the Supplementary Document). *Tukey’s* post hoc tests revealed *NR4A1* mRNA decreased significantly from PRE to POST (p<0.05) and from MID to POST (p<0.001) during both conditions (Fast and Fast+Ex). *Tukey’s* post hoc tests also demonstrated *NR1D1* mRNA decreased significantly from PRE to MID (p<0.001) and from PRE to POST (p<0.001) during both conditions (Fast and Fast+Ex).

### METAMEX

**Figure 5:**
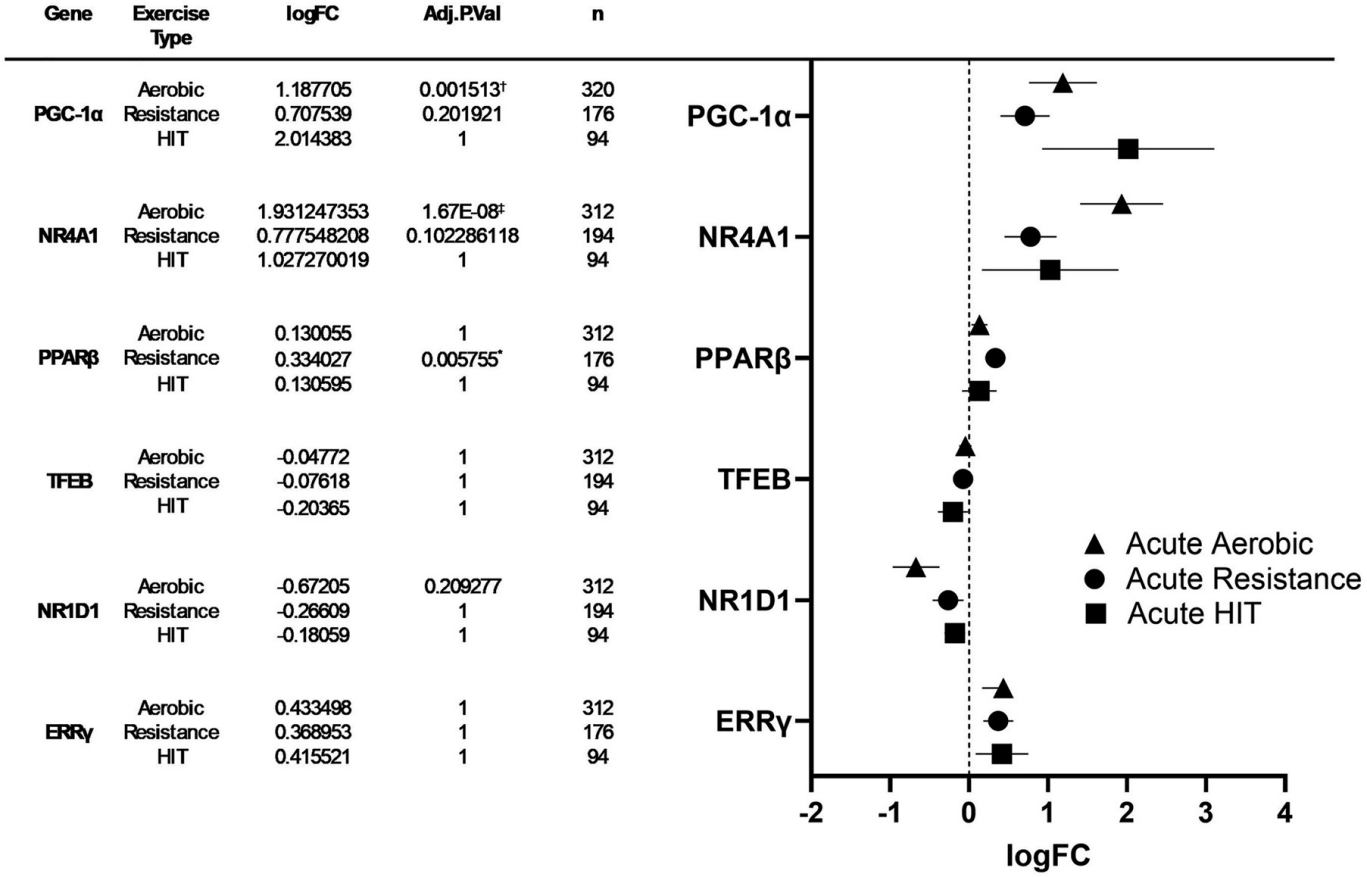
Left: Meta-analyzed expression change data of PGC-1α, NR4A1, PPARβ, TFEB, NR1D1, and ERRγ levels in human skeletal muscle after acute bouts of aerobic exercise, resistance exercise, and HIT exercise. Right: forest plot: log fold change and 95 % confidence intervals of PGC-1α, NR4A1, PPARβ, TFEB, NR1D1, and ESRRγ levels after an acute bout of aerobic exercise, resistance exercise, and HIT exercise. Symbols denote significant (*p≤0.05, †p≤0.001, ‡p≤0.0001) differences in mRNA expression. See worksheets S1–S7 in supplementary data for additional information.

We retrieved data from meta-analyzed human muscle gene expression using MetaMEx website (https://www.metamex.eu) [36] to determine the regulation of *PGC-1α* and of the selected novel regulators of mitochondrial biogenesis (*NR4A1*, *PPARβ*, *TFEB*, *NR1D1*, and *ERRγ*) in response to acute aerobic, resistance, and HIT exercise using the conditions described in the methods section above. Results from analyses including all available timepoints are presented in Figure 5 and Worksheet S5-S10 in the Supplementary Document.

Results of the time course analyses for changes in gene expression (*PGC-1α, NR4A1, PPARβ, TFEB, NR1D1, and ERRγ*) in response to exercise are presented in Table 2. Significant increases in gene expression compared to baseline were observed in the following genes and exercise time points: *PGC-1α* at 2–3 h and 4–6 h, *NR4A1* at 0–1 h and 2–3 h, *PPARβ* at 2–3 h and 4–6 h, and *ERRγ* at 4–6 h. Additionally, significant decreases were observed in *NR1D1* expression at 2–3 h and 4–6 h compared to baseline.

**Table 2:** MetaMEx time-course data for gene expression of *PGC-1α*, *NR4A1, PPARβ*, *TFEB, NR1D1, and ESRRγ*.

Time		PGC-1α	NR4A1	PPARβ	TFEB	NR1D1	ESRRγ
0–1 h vs. pre	Adjusted p value	1	p<0.001	1	1	1	1
	logFC	0.35	3.2	0.2	0.18	0.081	−0.099
2–3 h vs. pre	Adjusted p value	p<0.001	p<0.001	0.02	1	p<0.001	1
	logFC	2.9	2.8	0.67	−0.17	−1.4	0.11
4–6 h vs. pre	Adjusted p value	p<0.001	0.8	p<0.001	1	p<0.001	p<0.001
	logFC	1.2	0.52	0.33	−0.093	−0.62	0.83
24 h vs. pre	Adjusted p value	1	1	1	1	1	1
	logFC	−0.19	−1.3	−0.75	0.64	−1.6	−0.47
48 h vs. pre	Adjusted p value	1	1	1	1	1	1
	logFC	0.31	−1	−0.17	−0.97	−0.71	−0.081

## Discussion

The major novel findings of the current study are: 1) Acute HIIE increased *NR4A1* while decreasing *TFEB*, and 2) an 8 h fast (with or without additional energetic stress) decreased the expression of *NR4A1* and *NR1D1*. Meta analyses performed using MetaMEx suggest that *NR4A1* is robustly expressed following aerobic and resistance exercise in the early post exercise period (0–3 h) while small increases in *PPARβ* and *ERRγ* also occur post exercise (2–6 h). MetaMEx analyses also revealed a decrease in *NR1D1* expression in the early post exercise period (2–6 h).

### ERRγ

Estrogen related receptor γ (ERRγ; ESRRG) targets genes involved in mitochondrial biogenesis in rodent heart and skeletal muscle [11, 12]. Muscle-specific *ERRγ* overexpression augments mitochondrial protein content, enzyme activities, and/or respiration in mice [9, 10] even in the absence of *PGC-1α/β* [11]. *ERRγ* expression is increased in murine skeletal muscle following exercise [9, 11]. We failed to observe an increase in *ERRγ* mRNA following HIIE, but MetaMEx analyses demonstrated elevated expression levels 4–6 h post-exercise. It is possible that our biopsy time point post HIIE (3 h) prevented us from observing the delayed upregulation demonstrated by MetaMEx. The lack of change post HIIE in the current study agree with some [25] but not all [24] transcriptomic studies in human muscle and suggest the induction of *ERRγ* mRNA observed in exercised rodent muscle [9, 11] is not robustly conserved in humans.

To our knowledge, we are the first to examine the impacts of fasting on *ERRγ* expression in human muscle. No changes were observed in *ERRγ* mRNA following a short-term fasting period of 8 h (with or without 2 h of low-intensity upper-body exercise) suggesting that *ERRγ* expression changes may not occur during the initial hours of fasting in human muscle. The mechanisms underlying the regulation of *ERRγ* expression in human skeletal muscle (e.g., upstream signaling pathways) in response to exercise and fasting remain uncertain and should be studied in future human studies.

### PPARβ

Muscle-specific peroxisome proliferator-activated receptor β (aka PPARβ/PPARδ) overexpression promotes an oxidative phenotype and improves mitochondrial content [13, 14, 19]. Further, *PPARβ* knockdown/deletion reduces mitochondrial mRNA and/or protein [13]. *PPARβ* mRNA expression is sometimes [25], [26], [27], [28], [29, 42] but not always [24, 29] exercise-inducible in human skeletal muscle. Our results do little to resolve this controversy with *PPARβ* expression remaining unchanged following HIIE but MetaMEx analyses revealing small increases in *PPARβ* mRNA expression 2–6 h post exercise. The contradictory evidence in the literature and our current results highlights the need for robust investigations examining the exercise responsiveness of *PPARβ* and the role of *PPARβ* in human skeletal muscle function using alternative indices (e.g., PTMS, subcellular localization).

The effects of fasting on rodent skeletal muscle *PPARβ* expression is limited and contradictory with studies reporting fasting mediated increases [43, 44], decreases [34], or no change [45]. Our novel demonstration of unchanged *PPARβ* expression following 8 h of fasting (with or without additional energetic stress) suggests that fasting duration affects the expression of *PPARβ*, or that species-specific differences may exist in the regulation of this gene. Further research in human skeletal muscle is necessary to understand the exact mechanisms underlying the regulation of *PPARβ* in response to different durations and models (intermittent, time-restricted, etc.) of fasting.

### NR1D1

*NR1D1* modulates mitochondrial content and oxidative function via the activation of the LKB1-AMPK-SIRT1-PGC-1α signaling in mice [15]. *NR1D1* is also highly expressed in BDX mouse strains displaying upregulation of AMPK signaling and mitochondrial genes [5]. The knockdown of *NR1D1* in mice muscle increases the expression of atrophy related genes and reduces muscle fiber size [16]. Although endurance exercise (acute and chronic) increases skeletal muscle *NR1D1* levels in mice [5, 15, 46] – to our knowledge we are the first to examine the effects of exercise on *NR1D1* expression in human muscle. In contrast to results from mouse skeletal muscle we failed to observe an increase in *NR1D1* expression following HIIE and our MetaMex analysis revealed a decrease in *NR1D1* mRNA 2–6 h post exercise. Our results suggest that – unlike in murine muscle – exercise appears to either have no effect on or suppress the expression of *NR1D1* in human skeletal muscle.

We also observed a progressive decrease in *NR1D1* expression during an 8 h fast. We believe our observed suppression of *NR1D1* expression post fast is a novel observation in human skeletal muscle. Given our recent demonstration that fasting does not activate the AMPK-SIRT1-PGC-1α axis in human skeletal muscle [21], we speculate that the *NR1D1* suppression may contribute to the fasting response (i.e. the non-activation/suppression of the LKB1-AMPK-SIRT1-PGC-1α) in human muscle – a speculation that warrants future investigation.

### NR4A1

The NR4A family of orphan receptors – including NR4A1 (aka NUR77) – are implicated as important regulators of metabolic health [47]. Loss-of-function models identified *NR4A1* as a regulator of glucose metabolism in rodent skeletal muscle [18, 48], while *NR4A1* muscle-specific overexpression in mouse models improves glucose tolerance and fatty acid oxidative capacity [6, 49].

We observed increases in *NR4A1* mRNA following HIIE, a finding confirmed by robust increases following aerobic and resistance exercise in our MetaMEx analysis. These results agree with prior reports from gene array studies [24, 25, 30], [31], [32] and implicate *NR4A1* as a highly responsive, exercise inducible, gene. Given the powerful induction *NR4A1* expression by the adrenergic-cAMP-PKA-CREB pathway in rats [33], it seems likely that the upregulation of *NR4A1* observed in our study is mediated by the stimulation of the beta-adrenergic system [33, 48].

Interestingly, we observed a downregulation of *NR4A1* mRNA following an 8 h fast. Fasting increases the phosphorylation of CREB^Ser133^ [50] in human muscle. However, PKA activity in human skeletal muscle was unchanged following a 48 h fast [51]. Although it is tempting to speculate that the suppression of *NR4A1* may be associated with a downregulation/inhibition of adrenergic-cAMP-PKA-CREB pathway [33], this theory is untested in human skeletal muscle.

Our findings suggest that *NR4A1* mRNA is strongly induced by exercise and downregulated by 8 h of fasting. The functional implications of *NR4A1* mRNA regulation observed following exercise and fasting represent an important area for future study.

### TFEB

*TFEB* is implicated in the regulation of skeletal muscle mitochondrial quality control and/or biogenesis, lipid metabolism, and glucose homeostasis [8]. Nuclear localization, transcriptional activity, and expression of *TFEB* are increased following acute and chronic contractile activity in cellular and animal models [8, 17, 35, 52]. These effects are *PGC-1α* dependent in mouse muscle [17]. To our knowledge, only two transcriptomic studies have investigated the effects of exercise on *TFEB* expression in human skeletal muscle – both observing no change in response acute endurance exercise [24, 25]. *TFEB* expression remained unchanged following HIIE and MetaMEx analyses demonstrated no effect of exercise on *TFEB* expression. In contrast to cellular and animal studies [17, 52] our data suggest *TFEB* is not exercise-inducible in human skeletal muscle.

Starvation induces *TFEB* activation, nuclear translocation, and target gene transcription in *in vitro* models [7, 52]. Further, *TFEB* is upregulated in response to fasting in mouse skeletal muscle – potentially via an interaction with *PGC-1α* [35]. In contrast, we observed no change in *TFEB* expression following 8 h of fasting (with or without additional energetic stress), a discrepancy that may result from species-specific differences in the expression of *TFEB* in response to fasting or the short duration of fasting utilized in the current study. Further research is needed to clarify these differences and determine *TFEB* expression under different fasting and/or stress conditions in human skeletal muscle.

### Limitations and future directions

It is important to note that the current analyses were secondary and were not powered for examining the current gene set. Thus, it is likely that our results are limited by low statistical power and an elevated risk of both type I and II errors. The molecular response to exercise in skeletal muscle is incompletely understood due, in part, to limited time resolution provided by serial muscle biopsies. The current study is limited by a single biopsy post exercise, and only 2 biopsies during a relatively short-term fast. Future time course studies are needed to provide a complete time course of mRNA expression changes following exercise – as highlighted by our MetaMEx analysis – and during fasting periods lasting longer than 8 h. Although we have provided novel information on the impacts of exercise (HIIE) and fasting (8 h) on *ERRγ*, *PPARβ*, *NR1D1*, *NR4A1*, and *TFEB* in human skeletal muscle, the mechanisms underlying the regulation of these genes – and their importance in the in the mitochondrial biogenic response to exercise and fasting – remain poorly understood. Future studies exploring various forms of fasting (long term, intermittent, time-restricted, etc.) and a variety of exercise interventions (varying exercise intensities and training) are needed. Additionally, future studies in human skeletal muscle should measure *ERRγ*, *PPARβ*, *NR1D1*, *NR4A1*, and *TFEB* protein content and activity and assess the regulation of their respective upstream signaling and downstream gene sets following exercise and fasting. Finally, cross-sectional studies comparing the expression/content/activity across populations with high and low mitochondrial content/function (e.g. healthy vs. diseased and/or physically active vs. sedentary) may provide insight into the functional importance of *ERRγ*, *PPARβ*, *NR1D1*, *NR4A1*, and *TFEB* in human skeletal muscle.

### MetaMEx

Although MetaMEx provides an excellent tool to review data available in publicly available transcriptomics data sets, several limitations should be considered. First, there are many different options available when performing meta-analyses for individual genes (Sex, Age, Fitness, Weight, Muscle, and Health Status). Second, and similarly, there are many different time points that can be investigated (immediately post up to 96 h post). Third, the timeline analysis function of MetaMEx includes all data from healthy individuals and additional variables (sex, age, etc.) are not considered. In the current study we have included data from all healthy participants and all available time points in the meta-analyses results we report (Figure 5). Similarly, the timeline results presented (Table 2) reflect all healthy participants from all available exercise protocols. Thus, the MetaMEx data presented in the current study should be interpreted while considering the possibility that results are confounded by the inclusion of multiple different populations (e.g. young vs. old, lean vs. obese), different exercise protocols, a wide range of post exercise time points.

The ability to sort data by sex, age, fitness, weight, muscle, health status, and numerous post exercise time point makes MetaMEx a valuable tool in cross-referencing results and/or hypothesis generation. Reporting all the sub-analyses possible through MetaMEx is outside the scope of the current manuscript, however, we encourage interested readers to utilize the wide array of analyses possible at https://www.metamex.eu.

### Conclusions

The current study provides novel insight into the impact of acute energetic stress (exercise and short-term fasting) on *ERRγ*, *PPARβ*, *NR1D1*, *NR4A1*, and *TFEB* expression in human muscle. We observed increased *NR4A1* and *PGC-1α* following HIIE. MetaMEx demonstrated robust increase in *NR4A1* expression post exercise along with time point specific increases *PPARβ* and *ERRγ* and a decrease in *NR1D1*. An 8 h fast (with or without additional energetic stress) decreased the expression of *NR4A1* and *NR1D1* – an effect that may contribute to reductions in mitochondrial biogenesis and other energetically expensive processes during periods of restricted energy availability like fasting. The results of this study contribute to our understanding of the layered network of transcriptional regulators that contribute to the control of mitochondrial biogenesis in human skeletal muscle.

## Supplementary Material

Supplementary Material
